# What has become of the refugia hypothesis to explain biological diversity in Amazonia?

**DOI:** 10.1002/ece3.5051

**Published:** 2019-03-27

**Authors:** Daniel Gomes da Rocha, Igor L. Kaefer

**Affiliations:** ^1^ Department of Wildlife, Fish, and Conservation Biology, Graduate Group in Ecology University of California, Davis Davis California; ^2^ Grupo de Ecologia e Conservação de Felinos na Amazônia Instituto de Desenvolvimento Sustentável Mamirauá Tefé Brazil; ^3^ Instituto de Ciências Biológicas, Universidade Federal do Amazonas Manaus Brazil

**Keywords:** Amazon forest, diversification, evolutionary history, Pleistocene glaciation, speciation, vicariance

## Abstract

The spatial distribution of biodiversity and related processes is the core of Biogeography. Amazonia is the world's most diverse rainforest and the primary source of diversity to several Neotropical regions. The origins of such diversity continue to be an unresolved question in evolutionary biology. Among many competing hypotheses to explain the evolution of the Amazonian biodiversity, one stands out as the most influential: the refugia hypothesis by Jürgen Haffer. Here, we provide a chronological overview on how the refugia hypothesis evolved over the decades and how the criticism from different fields affected its acceptance. We conclude that the refugia hypothesis alone cannot explain the diversification of the complex Amazonian diversity, and perhaps it was not the most important diversification mechanism. However, the debate provoked by refugia has produced a great amount of knowledge on Amazonian climatic, geological, and evolutionary processes, as well as on species distributions, movements, and history.

## 1 INTRODUCTION

Why do some areas, such as the Amazonian forest, exhibit such rich diversity of species while other areas show such poor diversity? For centuries, naturalists and ecologists have been intrigued by the uneven spatial distribution of species diversity (Humboldt, 1808), and the reason behind this fact is one of the most prominent and long‐lasting debates in ecology. In 2005, *Science* Magazine celebrated its 125th anniversary with a special issue containing the most compelling questions that face current scientific inquiry. The question, “What Determines Species Diversity?” was featured in the top 25 (Pennisi, 2005). This probably reflects a general concern over the future of biodiversity: If we are to predict and mitigate the ongoing global biodiversity loss (Lovejoy & Nobre, 2018), we need to understand the processes responsible for creating and maintaining diversity (Ricklefs, 1987).

Many competing hypotheses dedicated to describing patterns of biodiversity spatial variation have been proposed (Hillebrand, 2004; MacArthur & Wilson, 1967; McCain, 2005; Mittelbach et al., 2001). Even though it is almost certain that environmental components (e.g., climate, energy, heterogeneity) affect diversity at local scale, the extent to which the evolutionary history of different areas influence their diversity is still controversial (Francis & Currie, 1998; Latham & Ricklefs, 1993; Ricklefs, Latham, & Qian, 1999; Svenning, Eiserhardt, Normand, Ordonez, & Sandel, 2015). The “historical hypotheses” can be tracked back to Wallace (1878), who invoked factors such as glaciations and ecosystem age to explain global biodiversity patterns. The importance of history's role on current species diversity patterns is based on the argument that all species have been generated in the past in specific locations, with differential speciation/extinction rates and dispersal limitations (McGlone, 1996). Therefore, it is expected that long‐term evolutionary history has left an imprint on the present spatial diversity patterns. Advocates of the historical hypothesis argue that local species diversity patterns can only be fully explained if larger spatial and temporal scales are taken into account (Ricklefs, 1987). However, evolutionary processes are less accessible to experimentation than ecological processes, making some historical hypothesis difficult to test (Francis & Currie, 1998; Ricklefs, 1987) or disprove (Magnusson, 1997), particularly in very diverse ecosystems.

Amazonia is the largest tropical forest in the world, representing over half of the planet's remaining rainforests and hosting a considerable part of the world's biodiversity (Myers, Mittermeier, Mittermeier, Fonseca, & Kent, 2000). More precisely, one in four known terrestrial species in the world lives in the Amazonian rainforests (Dirzo & Raven, 2003). The biodiversity of plant species in the Amazon basin, estimated to be as many as 50,000 plant species, is the highest on Earth (Hubbell et al., 2008). Amazonia usually has more plant species per area than other wet forests in Africa and Asia (Turner, 2001; but see Sullivan et al., 2017). Within Amazonia, the highest biodiversity areas are in the west, by the Andes, where many regions of high diversity and endemism for several taxa, particularly plants, birds, and mammals have been located (Rahbek & Graves, 2001; ter Steege et al., 2003; Tognelli & Kelt, 2004). Amazonia is not only the world's most diverse rainforest but also the primary source of diversity to other Neotropical regions (Antonelli et al., 2018).

If large‐scale species diversity pattern is intriguing, one might expect that the extremely diverse Amazonian forest plays an important role in solving this puzzle. In fact, Kanpp and Mallet (2003) stated that the origins of the Neotropical species are “the most mysterious of all” (p. 71). This is probably the reason why the “Amazon refugia hypothesis” became one of the most influential species diversity historical hypotheses ever proposed. It was first suggested by Haffer (1969) and has long been a paradigm for explaining biodiversity of Neotropical forests and the debate around it is still alive in the literature (Bush, 2017). The aim of this review was to assess how the acceptance of the Amazon refugia hypothesis has changed over time and to identify its main caveat, as well as the current amount of supporting evidence in the literature.

## 2 WHAT IS THE AMAZON REFUGIA HYPOTHESIS?

Around mid‐20th century, many biologists believed the only mechanism of species differentiation was geographic isolation of populations, which reduced gene flow. Therefore, it was challenging to explain how vast contiguous tropical forests could be so diverse. One common assumption was that extant differentiated forms that currently overlap distributions had been spatially separated in the past (Vuilleumier, 1971). In that context, Livingstone's (1967) and Moreau's (1963) studies provided evidence of considerable past climate and vegetational changes in Africa and the consequential effects on faunal diversification (Moreau, 1966). Jürgen Haffer's famous *Science* paper (1969) applied the same logic to propose that climate and vegetation changes were the main drivers for the Amazonian high species diversity. In his hypothesis explanation, Haffer postulated that

…during several dry climatic periods of the Pleistocene and post‐Pleistocene, the Amazonian forest was divided into a number of smaller forests which were isolated by tracts of open, nonforest vegetation. The remaining forest served as ‘refuge areas’ for numerous populations of forested animals, which deviated from one another during periods of geographic isolation. The isolated forests were again united during humid climatic periods when the intervening open country became once more forest‐covered, permitting the refuge‐area population to expand their ranges. (Haffer, 1969, p.131)

In other words, Haffer suggests that during the past glacial ages, Amazonia suffered several cycles of forest retraction and expansion, which favored speciation through population geographic isolation (vicariance). The “glacial refugia” was not a novel concept introduced by Haffer, but it had been recently proposed to explain species past distribution in Europe (Godwin Sir, 1975), Australia (Keast, 1961), and Africa (Moreau, 1966). Haffer used patterns of annual rainfall and present‐day avian distribution in Amazonia as evidence to identify probable geographic locations of nine forest refuges in Amazonian lowlands. The refugia hypothesis offered historical explanation to both the geographic distribution of closely related taxa and to the high species diversity pattern over large geographic scale within Amazonia. Although it was originally proposed for birds, the hypothesis was a working model that should apply for other forest‐dwelling species.

The Amazon refugia hypothesis was attractive because it was applicable for a wide range of taxa, established a clear relationship between biogeographic history and evolutionary mechanism, and created a diversity of questions for future investigators to explore. However, the hypothesis had some built‐in assumptions: (a) Species' geographic distribution data were valid for hypothesis generation; (b) Amazonia was drier during glaciation times due to decreases in the mean annual precipitation, and the past and current center of higher rainfall areas were coincident; (c) forested taxa experienced rapid speciation centered on ice ages; and most importantly, (d) allopatric speciation was the main mechanism that originated most Amazonian forest species (Bush & Oliveira, 2006).

## 3 THE CHRONOLOGY OF THE AMAZON REFUGIA HYPOTHESIS

During the years following Haffer's publication, the evidence supporting the refugia hypothesis mounted. Reconstructions of distributions of major vegetation types during the last glacial period, based on geomorphological data, indicated past existence of isolated forested areas corresponding in size and location to the proposed refugia (Ab'Saber, 1977). The hypothesis was reinforced by several biological studies on current diversity distribution of several animal groups, particularly birds (Haffer, 1978), lizards (Vanzolini & Williams, 1970), butterflies (Brown, 1977), scorpions (Lourenco, 1986), but also woody plants (Prance, 1973), and even diversity of languages in Amazonian tribes (Meggers, 1975). Those studies suggested particular regions with high diversity within Amazonia, with some spatial overlapping between regions across studies, corroborating the past existence of forest refugia in those areas.

The refugia hypothesis became popular and developed its own vocabulary. The term “refugia” expanded to include analogous terms such as “centers of dispersal,” “centers of distribution,” and “semi‐refuges” (Muller, 1973), and the word “hypothesis” was progressively replaced by “theory.” The paradox of the high species richness in the stable Amazonian region seemed solved by forest retraction/expansion in the glacial/interglacial dynamics (Vuilleumier, 1971), and conservation strategies overlapping protected areas, and refugia locations were suggested (Lovejoy, 1982; Perry, 1982). In the late 1970s, Simpson and Haffer (1978) compiled all the supporting evidence to consolidate the prominent position of the refugia hypothesis. As concluding remarks, they stated the following: “At present the evidence seems indisputable that much of the development of the modern Amazonian biota can be attributed to a succession of Pleistocene changes” (p. 514).

Criticism toward the forest refugia started to grow at the turn of the 1980s. Skeptical researchers doubted the actual evidence on the extreme forest cover changes during the Pleistocene and the mechanisms needed to cause them. Specifically, there were concerns on whether climatological drivers alone, particularly the predicted lower average temperature and rainfall, could cause massive savanna expansion over Amazonian forests during glacial times (Whitten, 1979). Climatologists also questioned the simplistic uniform decrease in the mean annual precipitation over the entire Amazonia region during glaciations, as predicted by the hypothesis (Whitten, 1979). Latter studies would confirm a high precipitation instability across the eastern and southern Amazonia during the Pleistocene, contrasting with much more stable climatic conditions in the west of the biome (Cheng et al., 2013; Mayle, 2000). Another source of criticism was on the circularity of using current species distribution patterns as the main evidence of the mechanism that generated these patterns (Endler, 1982a). Beven, Connor, and Beven (1984) argued that the observed spatial extent of overlap between sets of refugia proposed for different taxa, which was one of the main assumptions underpinning the hypothesis, was no different from that expected given random placement. Nelson, Ferreira, Dasilva, and Kawasaki (1990) suggested that high species diversity in some proposed refugia locations could be, at least for plants, due to sampling artifacts (but see De Oliveira & Daly, 1999). At this point, the theory seemed a premature idea and the call for independent geological, palynological, and paleoecological data increased (Connor, 1986).

Although there were some studies both supporting and contradicting the refugia hypothesis in the early 90s using mainly animals as models (Brumfield & Capparella, 1996; Froehlich, Supriatna, & Froehlich, 1991), the decade also witnessed the input of palynological studies to the reconstruction of past Amazonian vegetation cover, much to the detriment of the credibility of the refugia hypothesis. Pollen history obtained from sediment of lake and river bottoms in the lowland Amazonia indicated that forests continuously occupied regions believed to be savanna during the last glaciation maximum (Colinvaux, DeOliveira, Moreno, Miller, & Bush, 1996; Haberle & Maslin, 1999). Haffer argued that the long pollen data from Colinvaux et al. (1996), Colinvaux, De Oliveira, and Bush (2000) could not be used as evidence against the refuge existence because the pollen data were from the “Imerí refuge” region, which was predicted by the hypothesis to be forested during glacial periods (Haffer & Prance, 2001). However, as pollen and geological data accumulated, it reinforced the idea that Amazonian lowlands were forested throughout the late Quaternary and Tertiary periods (Colinvaux, Irion, Rasanen, Bush, & de Mello, 2001), with no evidence of forest fragmentation, except for some ecotone movements at the borders of the biome (Absy et al., 1991; Colinvaux et al., 2000; Fontes et al., 2017).

The pollen and geological data also have their own caveats. The first caveat is related to the spatial distribution of the lakes from which data comes from. To date, most of the studied lakes are located in the periphery of Amazonia or are from atypical higher elevation locations (as most lakes in lowland Amazonia are not that old; Cheng et al., 2013; Fontes et al., 2017). Hence, there are data gaps in central Amazonia concerning paleoecological data, which hampers the full understanding of forest fragmentation dynamics (if any) during the last glacial period. Besides spatial gaps, the second caveat refers to temporal gaps on the sedimentary records. For example, the Pata, Maicuru, and Carajás lake records contain temporal gaps with limited or no information (see D'Apolito, Absy, & Latrubesse, 2013), as well as the speleothem record studied by of Wang et al. (2017).

The uncertainty about the Pleistocene forest isolation argument created a gap for alternative hypotheses, such as the riverine hypothesis (Capparella, 1991), marine transgressions (Nores, 1999), and the disturbance vicariance hypothesis (Colinvaux, 1998). All those hypotheses evoked vicariant mechanisms other than climate‐driven vegetational changes to explain Amazonian diversity (see Haffer, 1997 for detailed description of those alternative hypotheses). The compelling set of evidence that savanna did not expand enough to create refugia during the Pleistocene made Haffer adjust his hypothesis to include climate‐driven vegetation change during the Neogene, Paleogene, or even before (Haffer, 1997). In his own words, “refuge theory does not refer to a particular time of speciation but to a particular mode of speciation” (Haffer, 2008, p. 934).

Forest fragmentation was the vicariance mechanism and the keystone of the refugia hypothesis. While some opponents of the refugia argued that other vicariance events (alternative historical hypothesis cited above) may have had a stronger influence than the vegetation changes on Amazonian species diversification (Cheviron, Hackett, & Capparella, 2005), others doubted whether any vicariant process was needed to explain Neotropical diversity (Kanpp & Mallet, 2003). The Amazon is a big enough space that long‐distance dispersal followed by allopatric isolation could readily occur and drive speciation (Dexter et al., 2017). Alternatively, Darwin (1958, p. 105) had suggested that parapatric speciation can be more important than allopatric speciation in generating diversity in vast, continuous habitats. Several studies have provided evidence and arguments that parapatric speciation played a role in the Neotropics (Brumfield & Capparella, 1996; Endler, 1982a; Ortiz, Lima, & Werneck, 2018; Smith, Wayne, Girman, & Bruford, 1997; Whinnett et al., 2005). This is possible because large areas have higher ecological diversity and support larger populations than isolated small areas and therefore usually have higher genetic variability subjected to speciation due to isolation by distance (Kanpp & Mallet, 2003). However, parapatric speciation proposals are often criticized because the mechanism of ecological divergence that contributes to reproductive isolation, which is a prerequisite for parapatric speciation, is usually unclear. Moreover, distinguishing the importance of parapatric from allopatric process can be problematic, as similar geographic congruence of species and species boundaries can sometimes be explained by both processes (Endler, 1982b).

Important studies were conducted to test predictions of the refugia hypothesis in the early 2000s. At this point, researchers were seeking to integrate spatial distribution and timing of the diversification of lineages to understand diversification patterns (Donoghue & Moore, 2003). One aspect of the refugia hypothesis that was challenged was the implicit prediction that divergence times should be coincident among taxa that share a common history. According to the refugia hypothesis, forest fragmentation should have simultaneously impacted vicariance of codistributed taxa. This was strongly refuted by the phylogenetic study of Whinnett et al. (2005) using Lepidoptera as a model. This study also corroborated the notion that parapatric differentiation may be an important driver in continuous Amazonian forests. Another important prediction of the refugia hypothesis is that forest animal populations must have experienced substantial demographic growth accompanying forest expansion during interglacial ages. Although one study found genetic evidence of substantial population growth accompanying habitat expansions for North American mammals, the study did not find similar evidence for Amazonian mammals after the last glacial period. (Lessa, Cook, & Patton, 2003). The refugia hypothesis would also predict a geographically restricted center of endemism, which is not corroborated by the extensive geographic distribution of several Amazonian tree species (Ter Steege et al., 2013).

Not only the synchrony of taxa diversification and the evidence of demographic expansion were challenged. By the 2000s, another pillar of the refugia hypothesis was under attack: the age of species diversification. Several independent molecular studies, with different animal taxa, indicated that times of species divergence preceded the Quaternary period (Boubli & Ditchfield, 2000; Clough & Summers, 2000; Glor, Vitt, & Larson, 2001; Hoorn et al., 2010; Moritz, Patton, Schneider, & Smith, 2000). Although a considerable amount of Amazonian tree diversity seems to have originated in the Pleistocene (Richardson, Pennington, Pennington, & Hollingsworth, 2001), most plant species diversity in the Neotropics was also proven to be ancient, with great diversification more than 50 Mya (Wilf, 2003; Wing et al., 2009). Even birds, which were used as a model in the original refugia‐hypothesis paper, began to diversify before the onset of the Pleistocene (Cheviron et al., 2005; Eberhard & Bermingham, 2005; Ribas, Gaban‐Lima, Miyaki, & Cracraft, 2005).

As most of the main pillars of the original Amazon refugia hypothesis had been questioned or falsified by the late 2000s, one might expect that the debate has ceased. Nevertheless, it has continued. Two years before his death, Haffer published a long paper responding to many of the palynological, geomorphological, and biological arguments against his hypothesis and that criticized authors who “directed critical comments at distorted caricatures of the Refuge hypothesis” (Haffer, 2008, p. 932). In the last decade, there have been many studies that provided evidence supporting (do Amaral et al., 2009; Fouquet et al., 2012; Garzon‐Orduna, Benetti‐Longhini, & Brower, 2014; Hubert et al., 2007; Mirabello & Conn, 2008; Pavan, Martins, Santos, Ditchfield, & Redondo, 2011; Ruiz‐Garcia et al., 2010; Thomas et al., 2012) and refuting (de Melo, Lima‐Ribeiro, Terribile, & Collevatti, 2016; Freycon, Krencker, Schwartz, Nasi, & Bonal, 2010; Fuchs, Chen, Johnson, & Mindell, 2011; Poelchau & Hamrick, 2013; Ribas, Aleixo, Nogueira, Miyaki, & Cracraft, 2012) the Pleistocene‐refugia hypothesis, consequently keeping the dispute alive.

## 4 FINAL REMARKS

The Amazon refugia hypothesis was an elegant and dominant paradigm that stimulated the debate of drivers of Neotropical diversity for half‐century. The hypothesis was largely influenced by the success of related temperate‐forest refugia hypotheses (Bennett, Tzedakis, & Willis, 1991) and reached its golden age when only indirect biogeographic evidence was available, but suffered severe opposition with the advance of the well‐dated paleoecological and molecular data. The two main current debates around the Amazon Refugia hypothesis are (a) the ancient versus Pleistocene diversification, and (b) the scarcity and incompleteness of existing paleoecology records that impede a satisfactory reconstruction of the paleovegetation basin‐wide.

The refugia hypothesis has changed over time, and the number, size, and location of the isolated refugia were loosened. The idea of an open savanna matrix, which was initially thought to separate refugia forests patches, was replaced by a mosaic of various types of savanna, dry forest, and other intermediate vegetation types (Sato & Cowling, 2017). The timeframe of the refugia hypothesis was also relaxed to “any period of the earth's history” as opposed to the original Pleistocene and post‐Pleistocene. The connectivity between refugia through riverine forests that were resistant to drier climates has also become acceptable. All these changes helped this hypothesis to survive in the face of new evidence.

During the past decades, there have been studies confirming and refuting almost every aspect of the refugia hypothesis. The most disputed aspect still seems to be about the fragmentation intensity that Amazonia experienced during the ice ages (Bush, 2017). Palynology and geology experts strongly disagree on the extent of past savanna‐like vegetation in the Amazonian region, based on available evidence. The only consensus is that Amazonian speciation and diversity are very complex (Antonelli et al., 2010; Hoorn et al., 2010), and that their elucidation still suffer from limited geographic and taxonomic sampling (Leite & Rogers, 2013).

It would be over simplistic to believe that such enormous diversity could be explained by evoking a single mechanism (Cheviron et al., 2005). Several studies have suggested that the Amazonian diversity appears to be the result of multiple factors with contribution of both allopatric and parapatric diversification mechanisms across different taxa (Whinnett et al., 2005). Vicariant processes including Andean uplift, riverine barriers, marine transgressions, and climatic‐driven vegetation shifts, as well as nonvicariant processes, such as range expansion, habitat gradients, and even domestication (Levis et al., 2017), played a role on the distributions of species. The Amazonian species variability is a result of all those processes acting independently, or in interplay, with variable intensities, at different times in different species (Antonelli et al., 2010; Noonan & Gaucher, 2005). That is probably why concordant evolutionary histories or simultaneous divergence times across Amazonian taxa are rarely found. Therefore, it is reasonable to say that most of the diversification of the Amazonian biota is not directly associated with the mechanism of the Pleistocene refuge hypothesis.

The history of Amazonian biodiversity continues to be an unresolved question in evolutionary biology (Antonelli et al., 2010). The forest refugia hypothesis cannot explain the origin and maintenance of the Amazonian diversity alone, and perhaps it was not the most important mechanism of species differentiation. From that perspective, the hypothesis has failed. However, not only the refugia hypothesis, but any simple unifying explanation for the Amazonian species diversity has also failed (Bush, 2005). Therefore, the refugia hypothesis, and many of the alternative hypotheses, still cannot be ruled out as relevant explanations for some aspects of the Amazonian diversity (Garzon‐Orduna et al., 2014). Even though there is no definitive or simple answer for the question concerning the origin of the incredible Amazonian biodiversity, the successive tests of the refugia hypothesis have generated much knowledge on Amazonian climatic, geological, and evolutionary process, as well as on species distributions, movements, and history (Bush & Oliveira, 2006). In this sense, it has been a successful hypothesis.

Understanding how Amazonian biodiversity has developed and identifying the related mechanisms has never been so urgent. For the past decades, Amazonia has been experiencing unprecedented threats, including persistent high deforestation rates, widespread use of fire, and global climate changes (Lovejoy & Nobre, 2018). It is expected that, with current deforestation trends, 40% of Amazon forests will be cleared and about a quarter of Amazon mammal species will lose more than 40% of their distribution by 2050 (Soares‐Filho et al., 2006). The deforestation might disrupt the hydrological cycle and tip the ecological point to flip forested to nonforest ecosystems in eastern, southern, and central Amazonia (Lovejoy & Nobre, 2018). A better understanding of the mechanisms behind the diversification processes might enable us to anticipate and mitigate ongoing biodiversity loss in the Amazonia.

## CONFLICT OF INTEREST

None declared.

## AUTHOR CONTRIBUTIONS

DGR conducted the literature review, and DGR and ILK wrote the paper.

## Data Availability

This is a review article, and we have not included any data in a publicly accessible repository.
